# Preclinical characterization of the CDK4/6 inhibitor LY2835219: in-vivo cell cycle-dependent/independent anti-tumor activities alone/in combination with gemcitabine

**DOI:** 10.1007/s10637-014-0120-7

**Published:** 2014-06-13

**Authors:** Lawrence M. Gelbert, Shufen Cai, Xi Lin, Concepcion Sanchez-Martinez, Miriam del Prado, Maria Jose Lallena, Raquel Torres, Rose T. Ajamie, Graham N. Wishart, Robert Steven Flack, Blake Lee Neubauer, Jamie Young, Edward M. Chan, Philip Iversen, Damien Cronier, Emiko Kreklau, Alfonso de Dios

**Affiliations:** 1 Eli Lilly and Company, Lilly Corporate Center, Indianapolis, IN 46285 USA; 2 Eli Lilly and Company, Alcobendas (Madrid), Madrid, Spain; 3 Eli Lilly and Company, Windlesham, UK; 4 Covance Laboratories, Greenfield, IN 46140 USA; 5 Present Address: Herman B. Wells Center for Pediatric Research and Indiana University Simon Cancer Center, Indiana University School of Medicin, Indianapolis, IN 46202 USA

**Keywords:** CDK4/6 inhibitor, Cell cycle, LY2835219, In vivo antitumor activity, Kinase inhibitor, Combination therapy

## Abstract

The G1 restriction point is critical for regulating the cell cycle and is controlled by the Rb pathway (CDK4/6-cyclin D1-Rb-p16/ink4a). This pathway is important because of its inactivation in a majority of human tumors. Transition through the restriction point requires phosphorylation of retinoblastoma protein (Rb) by CDK4/6, which are highly validated cancer drug targets. We present the identification and characterization of a potent CDK4/6 inhibitor, LY2835219. LY2835219 inhibits CDK4 and CDK6 with low nanomolar potency, inhibits Rb phosphorylation resulting in a G1 arrest and inhibition of proliferation, and its activity is specific for Rb-proficient cells. In vivo target inhibition studies show LY2835219 is a potent inhibitor of Rb phosphorylation, induces a complete cell cycle arrest and suppresses expression of several Rb-E2F-regulated proteins 24 hours after a single dose. Oral administration of LY2835219 inhibits tumor growth in human tumor xenografts representing different histologies in tumor-bearing mice. LY2835219 is effective and well tolerated when administered up to 56 days in immunodeficient mice without significant loss of body weight or tumor outgrowth. In calu-6 xenografts, LY2835219 in combination with gemcitabine enhanced in vivo antitumor activity without a G1 cell cycle arrest, but was associated with a reduction of ribonucleotide reductase expression. These results suggest LY2835219 may be used alone or in combination with standard-of-care cytotoxic therapy. In summary, we have identified a potent, orally active small-molecule inhibitor of CDK4/6 that is active in xenograft tumors. LY2835219 is currently in clinical development.

**Electronic supplementary material:**

The online version of this article (doi:10.1007/s10637-014-0120-7) contains supplementary material, which is available to authorized users.

## Introduction

Sustained proliferative capacity is a hallmark of cancer [1]. The cell cycle is the process by which mammalian cells regulate proliferation and has 4 functional phases: S phase when DNA replication occurs; M phase (mitosis): when DNA and cellular components are divided to form 2 daughter cells; the G2 phase between S and M when cells prepare for mitosis; and the G1 phase after mitosis and before phase S when cells commit and prepare for another round of DNA and cellular replication. The G1 restriction point (R) was originally described as the point where cell proliferation becomes independent of mitogens and growth factors [2], and the normal function of the restriction point is essential for maintaining control of cellular proliferation [3, 4]. The restriction point is controlled by the retinoblastoma pathway (CDK4/6-cyclin D1-Rb-p16/ink4a). The retinoblastoma protein (Rb) is a tumor suppressor that inhibits proliferation through binding to and suppressing the activity of the E2F family of transcription factors [5]. In early G1, when conditions are favorable for proliferation, D-type cyclin levels increase through transcriptional and posttranscriptional mechanisms [6]. Increased cyclin D drives the formation of active kinase heterodimers with CDK4 and CDK6 (CDK4/6) catalytic subunits. Active CDK4/6 then phosphorylates Rb, partially relieving suppression of E2F to allow expression of genes required for passage through the restriction point [7, 8]. This includes expression of cyclin E, which activates another kinase (CDK2) leading to hyperphosphorylation of Rb, fully releasing the suppression of E2F: allowing cells to exit the G1 phase and initiate DNA replication. Additional restriction point control occurs through the action of the endogenous CDK inhibitors p16/ink4a and p21cip1; p16/ink4a blocks the binding of D-type cyclins to CDK4/6; CDK4/6 cyclin D complexes are stabilized by p21cip1, also sequestering it and preventing the inhibition of CDK2/cyclin E [4]. Phosphorylation of Rb by CDK4/6 also leads to transcription of genes involved in cell cycle-independent activities including signal transduction, DNA repair transcriptional control, and mRNA processing [9]. CDK4/6 have also recently been shown to phosphorylate other proteins, including FOXM1 and E2F1 [10, 11], which modulate additional cellular processes that are cell cycle -independent.

The central role of the Rb pathway in controlling cellular proliferation is highlighted by its frequent dysregulation in human cancer. Aberrant expression of D-type cyclins results in activation of CDK4/6, allowing cells to circumvent the restriction point. In mantle cell lymphoma (MCL), cyclin D1 is upregulated by the (11;14) chromosomal translocation seen in this tumor, and cyclin D1 is overexpressed in many breast, head and neck, prostate, and melanoma tumors [12, 13]. D-type cyclins have also been shown to be downstream effectors of Flt3 in acute myeloid leukemia (AML) [14]. CDK4 is amplified or overexpressed in several tumors, including soft tissue sarcomas, glioblastoma, and melanoma [15–18]. CDK6 has also been shown to be overexpressed in tumors [4]. Both p16/ink4a and Rb act as tumor suppressors and are found to be functionally inactivated in numerous tumor types [19, 20].

While CDK4/6 are considered highly validated targets for therapeutic intervention, progress toward development of inhibitors for these kinases has been limited by issues with potency, selectivity, and poor pharmacological/physiochemical properties. Examples of these initial efforts include flavopiridol, UCN-01, and CYC202 (roscovitine) [21]. Fry et al. recently reported a CDK4/6 inhibitor, PD 0332991, possessing improved potency and selectivity over previous inhibitors [22]. Here, we report the identification of LY2835219, a potent inhibitor of CDK4/6 that inhibits phosphorylation of Rb and induces a G1 cell cycle arrest in Rb-proficient tumor cells in vitro. In vivo, LY2835219 inhibits phosphorylation of Rb by CDK4/6 causing a G1 arrest resulting in antitumor activity in human tumor xenograft models. Physiochemical and pharmacological properties of the compound allow for oral administration, and no tumor outgrowth or significant adverse activities were seen with prolonged administration. When LY2835219 is used in combination with gemcitabine, enhanced inhibition of tumor growth is seen and is associated with a reduction of ribonucleotide reductase (RR) expression without a significant inhibition of Rb phosphorylation, suggesting a cell cycle independent mechanism of action for LY2835219 when combined with gemcitabine.

## Materials and methods

### Chemicals

LY2835219 [5-(4-Ethyl-piperazin-1-ylmethyl)-pyridin-2-yl]-[5-fluoro-4-(7-fluoro-3-isopropyl-2-methyl-3H-benzoimidazol-5-yl)-pyrimidin-2-yl]-amine, PD0332991 [6-Acetyl-8-cyclopentyl-5-methyl-2-[[5-(1-piperazinyl)-2-pyridinyl] amino] pyrido[2,3-d] pyrimidin-7 (8H)-one] and gemcitabine [2′,2′-difluorodeoxycytidine hydrochloric salt] were synthesized and characterized for purity and identity at Lilly Research laboratories. Data for LY2835219 and PD0332991 described herein were obtained using the methanesulfonate salt of each compound.

### Cell lines

All cell lines were obtained from American Type Culture Collection and maintained using the recommended culture conditions. Colo-205 was obtained in April 2005. MV-4-11 was obtained in August 2005. MDA-MB-231 was obtained in February 2005. MDA-MB-468 was obtained in August 2008. U2OS was obtained in March 2009. U-87 MG was obtained in May 2004. NCI-H460 was obtained in February 2005. Calu-6 was obtained in February 2005 and Jeko-1 in November 2009. MCF10A was obtained in September 2011. MDA-MB-361 was obtained in November 2013. Cell line authenticity was confirmed by DNA fingerprinting (IDEXX RADIL).

### Enzymatic profiling

Details for enzymatic assays are provided in the supplementary materials. Briefly, CDK4 and CDK6 activity was determined by radiometric-filter binding assay using a c-terminus fragment of the human Rb protein (containing amino acids 773 to 928, Millipore) as a substrate. Human CDK4/cyclin D1 and CDK6/cyclin D1 complexes were expressed in insect cells and purified as described (ProQinase). Test compounds were serially diluted 1:3 in 20 % DMSO to create a 10-point curve at a starting concentration of 20 μM. Twenty percent DMSO buffer alone without test compound was employed as a control; 500 mM EDTA was used to determine the level of background in the absence of enzyme activity. A 4-parameter logistic curve fit is used to generate the IC_50_ values using ActivityBase™ software (IDBS). For kinetic analysis, a range of ATP concentrations was used and Ki for both CDK4/cyclin D1 and CDK6/cyclin D1 complexes was determined by fitting to the Michaelis-Menten equation for a competitive inhibitor using GraphPad Prism. Detailed methods are found in the supplemental materials.

### Cell assays

Details for the cellular assays are provided in the supplemental materials. Briefly, image-based assays (Acumen Explorer, TPP LabTech) were used for compound screening. Cells were seeded in 96-well dishes, allowed to attach overnight and then treated with test compounds for 24 h. After treatment, the cells were washed, fixed and stained with antibodies to detect phosphoproteins and Propidium Iodide to monitor DNA content. The plates were then analyzed on an Acumen Explorer (TTP Labtech). The IC_50_ and EC_50_ values are determined by curve fitting to a 4-parameter logistic for each output using ACTIVITY BASE™ software.

Flow cytometry was also used to determine cell cycle distribution in cell lines, as described in the supplemental materials.

### Western blot analysis

Cells or tissues were lysed in a buffer containing 1 % SDS in water, protease (Roche Applied Science) and phosphatase (Sigma Aldrich) inhibitors. Following lysis, the samples were heated at 95 °C for 10 min, passed through a 25G needle to shear genomic DNA, and briefly sonicated. Proteins (10–20 μg per lane) were resolved SDS-PAGE on 4–20 % Tris-glycine gels, transferred to nitrocellulose membranes and blocked for 1 h at room temperature in 5 % dry milk in TBST (137 mM sodium chloride, 20 mM Tris, 0.05 % Tween-20). The membranes were then incubated overnight at 4 °C in primary antibody diluted in 5 % BSA/TBST, a list of primary antibodies used is provided in the supplemental materials. Membranes were then washed in TBST, incubated 1 h at room temperature with the appropriate horseradish peroxidase conjugated secondary antibody (Amersham ECL) in 3 % milk/TBST, washed with TBST and developed using chemiluminescent substrate (SuperSignal Western, Pierce). Western blot band intensity was determined using a BioRad VersaDoc Molecular Imager with TotalLab software (BioSystematica). The percent change in band intensity was calculated using vehicle-treated sample bands as 100 %. One-way ANOVA statistical analysis of the data was performed using JMP software (SAS Institute).

### Tumor xenograft studies

All animal studies were performed in accordance with American Association for Laboratory Animal Care institutional guidelines, and all protocols were approved by the Eli Lilly and Company Animal Care and Use Committee. Tumor cell lines were grown, harvested, and resuspended in a 1:1 mixture of serum-free media and matrigel (BD Biosciences), and 5 × 10^6^ cells were injected subcutaneously in the rear flank of 5-to-6-week-old CD1 *nu/nu* female mice (Charles River Laboratories for MV4-11, mice from Harlan Laboratories for others). Tumor volume was estimated by using the formula: vol = l × w^2^ × 0.536, where one and w are perpendicular measured diameters, and one is greater than or equal to w.

When the mean tumor volume was approximately 150–300 mm^3^, animals were randomized by tumor volume and the compound was administered. LY2835219 was formulated in 1 % hydroxyethyl cellulose + 0.1 % antifoam in 25 mM PB pH 2 and administered orally by gavage (final volume 0.2 mL) at the indicated dose and schedule. Gemcitabine was formulated in saline and administered by intraperitoneal injection. Tumor volume and body weight were measured twice weekly. When tumors were collected for biomarker analysis, the animals were asphyxiated with CO_2_ and the xenograft tumors excised, flash frozen in liquid nitrogen, and stored at −80 ºC until analyzed. For analysis, tumor volume data were transformed to a log scale to equalize variance across time and treatment groups. The log volume data were analyzed with a 2-way repeated measures analysis of variance by time and treatment using the MIXED procedure in SAS software (version 9.2). The correlation model for the repeated measures was spatial power. Treated groups are compared to the control group at each time point.

## Results

### Identification of LY2835219

**Fig. 1:**
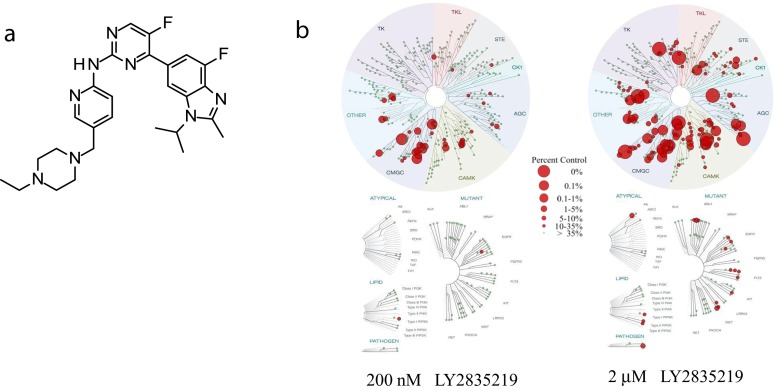
**a** Structure of LY2835219. **b** KINOME*scan* dendogram for biochemical kinase selectivity profile against 456 kinases. single point binding at 200 nM (*left*) and 2 μM (*right*) LY2835219

Compound screening identified the 2-Anilino-2,4-Pyrimidine-[5-Benzimidazole] scaffold as potent inhibitors of CDK4/cyclin D1 and CDK6/cyclin D1. The scaffold was subsequently optimized through extensive structure–activity relationship studies with the aid of structure-based design, by biochemical screening against a small panel of kinases to improve potency and selectivity, and with a colo-205 cell high content imaging assay monitoring inhibition of Rb phosphorylation, cell cycle distribution, and cell number to assess cellular inhibition of CDK4/6. Compounds with potent cellular activity were subsequently tested for cell cycle activity with special focus in optimizing for G1 arrest in vitro and in vivo (see discussion below), in vivo inhibition of Rb phosphorylation and pharmacokinetic properties. Superior compounds in terms of physicochemical and pharmacokinetic properties were then tested for in vivo antitumor activity against xenograft tumors in immunodeficient mice. LY2835219 was selected for its potent biological activities and optimal pharmacological properties within this chemical series (Fig. 1a). All preclinical characterization was performed with the methanesulfonate salt.

In biochemical assays, LY2835219 inhibits CDK4/cyclin D1 and CDK6/cyclin D1 with IC_50_ = 2 nmol/L and 10 nmol/L, respectively, and shows selectivity over closely related cell cycle kinases (Table 1). Precise Ki ^ATP^ constants were determined through kinetic studies, showing Ki^ATP^ = 0.6 nmol/L and 2.4 nmol/L for CDK4/cyclin D1 and CDK6/cyclin D1, respectively, indicating LY2835219 is a competitive ATP inhibitor. Kinase selectivity was further evaluated in a panel of 100 protein kinases (Millipore/UBI). Single point concentration assays were followed by IC_50_ determination for the most active kinases (Table 1). LY2835219 was also later profiled against 456 kinases (KINOME*scan*, DiscoverRx) for single point binding at 200 nM and 2 μM concentrations (Fig. 1b). When tested at approximately 200 fold concentration over the Ki for CDK4, the observed binding profile was significantly concentrated in the CGMC family of kinases that comprises cyclin-dependent kinases and MAPK kinases (Fig. 1b, left panel), which is consistent with the IC_50_s obtained from the original screening of 100 kinases. Out of all these biochemical profiling activities, it is particularly important to observe approximately 2–3 orders of magnitude in measured IC_50_s in biochemical kinase selectivity against CDK1/cyclin B1, CDK2/cyclin E, and CDK7/Mat1/cyclin H. Complete lack of activity against other important cell-cycle related kinases for which inhibition could also lead to potentially confounding cell cycle arrest in G2/M such as Aurora A, B, and PLK1 was noted. Activity against CDK9/cyclin T1 was seen (IC_50_ = 57 nmol/L); but did not translate to significant cellular CDK9 activity (inhibition of RNA polymerase C-terminal domain [Ser2] phosphorylation (pCTD) or a G2/M arrest in U2OS cells, Figure S1 and Table S1, Supplemental materials), possibly due to compensation by CDK7 [23]. Activity was also seen against PIM1 (IC_50_ = 50 nmol/L) and to a lesser extent against PIM2 (IC_50_ = 3.4 μmol/L, Table 1).

**Table 1:** LY2835219 biochemical and cellular profiling

LY2835219	
Biochemical profiling^a^	Ki [ATP] (nmol/L)
CDK4/cyclinD1	0.6 ± 0.3 (*n* = 2)
CDK6/cyclinD1	2.4 ± 1.2 (*n* = 2)
	IC50 (nmol/L)^b^
CDK4/cyclinD1	2.0 ± 0.4 (*n* = 5)
CDK6/cyclinD1	9.9 (*n* = 1)
CDK1/cyclinB1	1627 ± 666 (*n* = 5)
CDK2/cyclinE	504 ± 298 (*n* = 3)
CDK9/cyclinT1	57 ± 42 (*n* = 4)
CDK7/Mat1/cyclin H1	3910 ± 2410 (*n* = 4)
PIM1	50 (*n* = 1)
PIM2	3400 (*n* = 1)
bRaf	6330 (*n* = 2)
Aurora A	>20000 (*n* = 1)
Aurora B	>20000 (*n* = 1)
PLK1	>20000 (*n* = 1)
PLK3	>20000 (*n* = 1)
ERK1	>20000 (*n* = 1)
cRaf	>20000 (*n* = 2)
AKT1	>20000 (*n* = 1)
HIPK2	31 (*n* = 1)^c^
DYRK2	61 (*n* = 1)^c^
CK2	117 (*n* = 1)^c^
GSK3b	192 (*n* = 1)
CDK5/p35	287 (*n* = 1)^c^
CDK5/p25	355 (*n* = 1)^c^
JNK3	389 (*n* = 1)^c^
FLT3 (D835Y)	403 (*n* = 1)^c^
DRAK1	659 (*n* = 1)^c^
TRKA	1030 (*n* = 1)^c^
FLT3	3960 (*n* = 1)^c^
Cellular profiling	IC50 (nmol/L)^d^
pRb inh. (pSer780) colo-205	120 ± 36 (*n* = 6)
G1 arrest colo-205	72 ± 31 (*n* = 7, EC_50_)
pRb inh. (pSer780) MDA-MB-361	60 ± 40 (*n* = 3)^e^
G1 arrest MDA-MB-361	20 ± 13 (*n* = 3, EC_50_)^e^
Cell viability MDA-MB-361	90 ± 31 (*n* = 3)^e^
pCTD inh. (pSer2) U2OS	3510 ± 1560 (*n* = 4)
pHH1 inh. Calu6	17400 ± 4000 (*n* = 4)
pERK inh. HCT116	>20000 (*n* = 2)
pERK inh. A375	>20000 (*n* = 2)

(*a*) unless otherwise indicated, all data was generated internally at Eli Lilly and company. All human kinases. (*b*) for n > 1, average of independent determinations ± standard deviation. (*c*) IC_50_ generated at millipore (UBI) kinase panel for kinases >90 % inhibition at 200 nM concentration of inhibitor. (*d*) geometric mean for n > 1 determinations ± standard deviation. (*e*) determined after 6 days of incubation due to long doubling times for MDA-MB-361

### Cellular activity of LY2835219

**Fig. 2:**
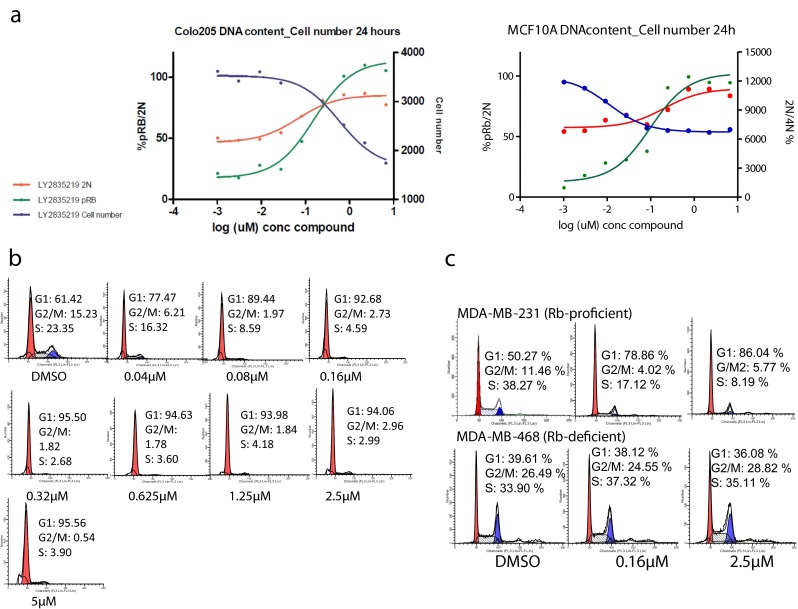
**a** Activity of LY2835219 in colo-205 and MCF10A cells using a multiplexed assay measuring p-Rb serine 780 (*green*), cells in G1 (2 N DNA content, *red*) and total cell number (*black*). after 24-hour exposure LY2835219 shows a dose-dependent inhibition of p-Rb (ser780) and a corresponding sustained G1 arrest. **b** MV4-11 acute myelocytic leukemia (AML) cells treated with increasing concentrations of LY2835219 for 24 h and cell cycle activity profiled by flow cytometry. **c** cell cycle activity of LY2835219 in cells with and without functional Rb. LY2835219 gives a G1 cell cycle arrest in Rb-proficient MDA-MB-231 breast cancer cells, but not in Rb-deficient MDA-MB-468 breast cancer cells

Rb Phosphorylation by CDK4/6 is required for cells to proceed through the G1 restriction point [8]. Therefore, inhibition of CDK4/6 will prevent Rb phosphorylation, arrest cells in G1 at the restriction point, and inhibit proliferation. Several complementary cellular assays were used to monitor Rb phosphorylation, cell cycle status, and proliferation. Cellular activity was assessed in tumor cell lines of different histologies, Rb status and relevant oncogenic alterations (Supplemental materials Table S2). Cellular inhibition of CDK4/6 was determined by monitoring Rb phosphorylation at serine 780 (p-Rb), which is specific for CDK4/6 [24]. Cell cycle status was monitored by measuring cellular DNA content and/or western blot analysis for cell cycle-specific markers; proliferation was assessed by changes in cell number. Unless otherwise noted, all cell assays were performed with compound treatment for 24 h, approximately one cell cycle, to observe changes in cell cycle distribution. Colo-205 colorectal cells treated with LY2835219 showed a concentration-dependent inhibition of p-Rb and a corresponding arrest of cells in G1 (2 N DNA content), resulting in a decreased number of cells due to inhibition of proliferation. The IC_50_ for p-Rb in colo-205 cells was 120 nmol/L; the EC_50_ for accumulation of cells with 2 N DNA content was 72 nmol/L (Fig. 2a). Inhibition of p-Rb and the G1 arrest was maintained at compound concentrations up to 6,000 nmol/L (approximately 50× the EC_50_ for p-Rb and 80× the EC_50_ for G1 arrest) consistent with no off-target cell-cycle activity within that concentration range. Similar results were observed for several breast cancer cell lines, as for example MDA-MB-361 (Table 1) and MCF10A. In MCF10A and after 24 h of compound incubation, consistent inhibition of pRb and sustained G1 arrest was observed (Fig. 2a).

The ability of LY2835219 to inhibit p-Rb and induce a G1 arrest was also confirmed in MV4-11 AML cells. Maximum G1 arrest was seen at concentrations ≥320 nmol/L (Fig. 2b). Western blot analysis was performed using the three cell-cycle-specific markers: p-Rb, a marker for CDK4/6 inhibition and a phenotypic marker for cells in G1; topisomerase II alpha (TopoIIα), a marker for cells in S phase [25]; and phospho-histone H3 serine 10 (pHH3), a marker for cells in M phase [26]. A sustained G1 arrest is indicated by strong inhibition of all markers. LY2835219 inhibits all 3 markers, consistent with cells arrested in G1 (Supplemental materials Figure S2). Inhibition of PIM1 kinase was also observed in MV4-11 cells as indicated by a dose-dependent inhibition of phosphorylation of several PIM substrates including pBAD (Ser112) and 4E-BP1 (Thr37/46) (Supplemental materials Figure S3). Similar to colo-205 and MCF10A cells, the G1 arrest in MV4-11 cells was sustained at high compound concentrations (up to 5,000 nmol/L).

Functional Rb protein is required for CDK4/6 to regulate the G1 restriction point; the mechanism of action for LY2835219 was further defined in breast cancer cell lines with and without functional Rb [27]. LY2835219 induced a G1 arrest in Rb-proficient MDA-MB-231 breast cancer cells, whereas no such effect was seen in Rb-deficient MDA-MB-468 breast cancer cells at compound concentrations up to 2,500 nmol/L (Fig. 2c). The data are consistent with CDK4/6 inhibition and with the hypothesis that G1 arrest induced by LY2835219 requires functional Rb.

### LY2835219 inhibits Rb phosphorylation by CDK4/6 causing a sustained G1 arrest in vivo

**Fig. 3:**
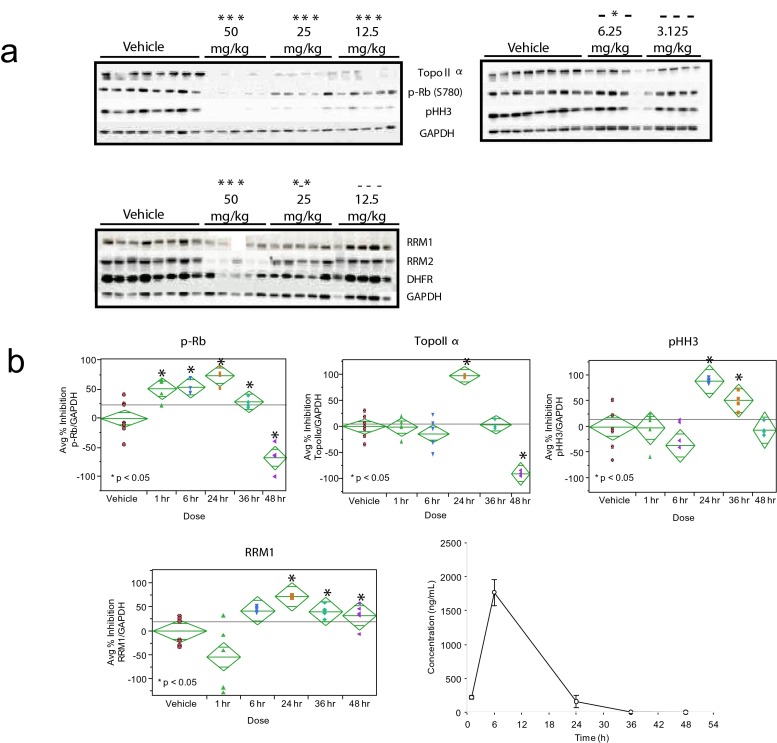
In vivo target inhibition by LY2835219. **a** tumors harvested 24 h after dosing for analysis with the markers topoIIα (S phase-specific), p-Rb (pRb ser 780, G1-specific), pHH3 (M-specific), and the Rb/E2F regulated proteins DHFR, RRM1 and RRM2. GAPDH was used as a loading control. statistical significance (*p* <0.05) is indicated above each dose by an asterisk for pRb, Topollα, and pHH3, respectively. **b** time course of LY2835219. tumor-bearing animals were dosed orally with 50 mg/kg of LY2835219 and tumors harvested at the indicated times (*n* = 5 per treatment, *n* = 8 for vehicle). the percent change in band intensity was calculated using vehicle-treated bands as 100 %. mean (±SD) plasma concentrations of the compound are shown in the lower right panel

Pharmacokinetic and pharmacodynamic properties of LY2835219 were assessed in mice bearing colo-205 human xenografts 24 h after oral dosing to allow for the observation of cell cycle effects. In vivo inhibition of p-Rb was dose-dependent and correlated well with inhibition of TopoIIα and pHH3 with all three markers significantly inhibited at doses of 12.5 mg/kg and higher (Fig. 3a). Time course studies showed maximum inhibition occurred 24 h after administration (Fig. 3b). The cell cycle arrest was reversible, with cells released back into the cell cycle in a sequence predicted by a G1 arrest. Thirty-six hours after dosing, inhibition of CDK4/6 was released and levels of p-Rb returned to baseline as Rb was again phosphorylated by CDK4/6. TopoIIα levels also increased as cells cycled into S phase, but pHH3 remained inhibited as cells released from G1 had not yet reached M phase. By 48 h, pHH3 returned to baseline, consistent with cells entering M phase; levels of p-Rb and TopoIIα rebounded above baseline, indicating a partial synchronization of tumor cells. Pharmacokinetic analysis showed plasma levels of LY2835219 peaked before inhibition of all markers, indicating the cell-cycle arrest can occur after maximal plasma exposure (Fig. 3b).

In addition to the cell cycle markers, we assessed the modulation of several Rb/E2F-regulated proteins that are the targets of cytotoxic drugs [28]. In colo-205 cells, DHFR, RRM1 and RRM2 were shown to be inhibited in a dose- and time-dependent manner similar to the cell cycle markers (Fig. 3a, b).

### In vivo antitumor activity of LY2835219 alone and in combination with gemcitabine

**Fig. 4:**
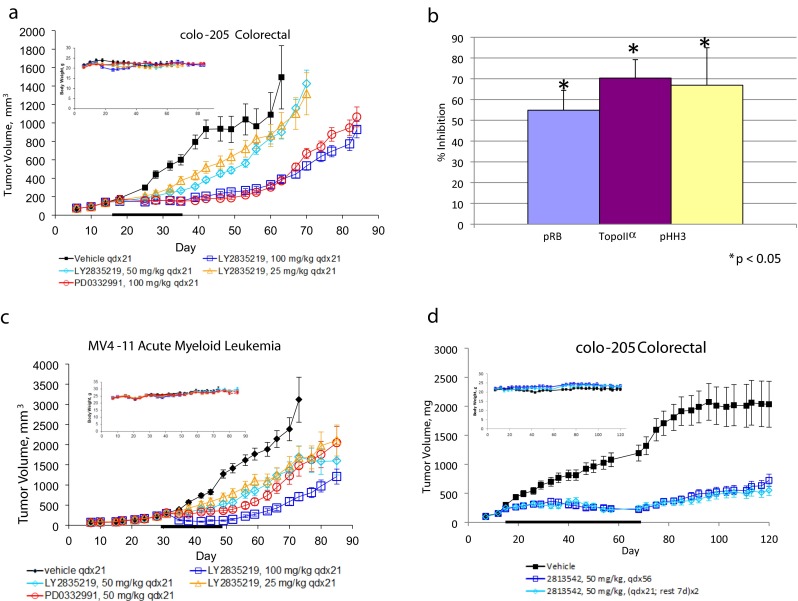
In vivo antitumor activity of LY2835219 in subcutaneous human tumor xenografts. Tumors were implanted in the rear flank of athymic mice and randomized for treatment when the mean tumor volume reached 150 to 200 mm^3^. LY2835219 and PD0332991 were administered at the indicated dose and schedule. treatment period is indicated by the horizontal black bar along the X-axis, body weight shown for each experiment is shown in the upper left corner. **a** effect of 25, 50, or 100 mg/kg of LY2835219 and 100 mg/kg PD0332991 on colo-205 xenografts. **b** inhibition of cell cycle markers in colo-205 xenografts at the end of treatment with 100 mg/kg LY2835219. **c** effect of 25, 50, or 100 mg/kg LY2835219 and 50 mg/kg PD0332991 in MV4-11 xenografts. **d** LY2835219 inhibits tumor growth and is well tolerated in mice bearing colo-205 xenografts when dosed 56 days with 50 mg/kg continuously or intermittently

In vivo antitumor activity of LY2835219 was assessed in subcutaneous human tumor xenografts. LY2835219 significantly inhibited the growth of colo-205 xenografts; doses up to 100 mg/kg were well tolerated with no loss of body weight or other signs of toxicity during or after treatment (Fig. 4a). The inhibition of tumor growth at the end of treatment was dose-dependent from 25 to 100 mg/kg doses (Fig. 4a, Table 2). Tumors from additional animals treated with 100 mg/kg LY2835219 for 21 days were harvested and cell cycle status determined by western blot; significant inhibition of p-Rb, TopoIIα and pHH3 was seen (Fig. 4b). This was consistent with the antitumor activity of LY2835219, attributable to inhibition of Rb phosphorylation by CDK4/6, resulting in a G1 cell cycle arrest. Antitumor activity was seen in other xenograft tumors of different histologies including MV4-11 AML, U87 MG glioblastoma, and H460 lung (Fig. 4c, Table 2, see also Table S2 Supplemental materials). LY2835219 also inhibited colo-205 tumor growth at a dose of 12.5 mg/kg every day, which corresponds with the minimum dose required to inhibit the cell cycle markers, and when administered every other day at 50 mg/kg (Table 2). The antitumor activity of LY2835219 was similar to another CDK4/6 inhibitor in these subcutaneous xenografts (PD0332991, Fig. 4a–c, Table 2); further indicating LY2835219 is specifically inhibiting CDK4/6.

**Table 2:** Antitumor activity of LY2835219 in subcutaneous human tumor xenografts

Xenograft model	Compound dose	Percent delta T/C, percent regression	*P*-Value	Day calculated
Colo-205 colorectal	100 mg/kg qdx21	0	<0.001	day 35
	50 mg/kg qdx21	28	<0.001	day 35
	25 mg/kg qdx21	52	<0.001	day 35
	PD0332991, 100 mg/kg qdx21	−1	<0.001	day 35
MV4-11 AML	100 mg/kg qdx21	−48	<0.001	day 49
	50 mg/kg qdx21	30	<0.001	day 49
	25 mg/kg qdx21	45	0.008	day 49
	PD0332991, 50 mg/kg qdx21	16	<0.001	day 49
U87 MG glioblastoma	100 mg/kg qdx21	1	<0.001	day 31
	50 mg/kg qdx21	33	<0.001	day 31
	25 mg/kg qdx21	49	0.001	day 31
	PD0332991, 50 mg/kg qdx21	26	<0.001	day 31
H460 lung	100 mg/kg qdx21	36	<0.001	day 35
	50 mg/kg qdx21	60	<0.001	day 35
	25 mg/kg qdx21	84	NS	day 35
	PD0332991, 50 mg/kg qdx21	60	<0.001	day 35
Colo-205 colorectal	12.5 mg/kg qd	53	0.087	day 39
	50 mg/kg q2d	27	0.003	day 39
	50 mg/kg qd	0	<0.001	day 39
	PD0332991, 50 mg/kg	7	<0.001	day 39
Calu-6 lung	50 mg/kg, qdx10	42	0.007	day 20
	50 mg/kg, qdx22	48	<0.001	day 34
Jeko1 MCL	100 mg/kg	10	<0.001	day 45
	PD0332991, 100 mg/kg	12	<0.001	day 45

100 % if treated volume = control volume

0 % if treated volume = baseline volume

−100 % if treated volume = zero

with linear interpolation between these points

To assess the long-term antitumor activity and safety of LY2835219, colo-205 xenograft-bearing mice were treated for 56 days with continuous and intermittent dosing schedules (56 days continuously or 2 cycles of 3 weeks on/1 week rest, Fig. 4d). Both schedules produced a similar robust inhibition of tumor growth and were well tolerated with no loss of body weight. Hematologic and histopathologic evaluations from treated animals on days 41 and 69 showed no significant changes in intestinal tissue (jejunum, ileum, cecum). Some hematologic changes were observed on both schedules (including decreases in red cell mass and moderately reduced lymphocyte count) but were not dose-limiting. Acquired drug resistance can occur due to prolonged treatment with kinase inhibitors [29]. No tumor outgrowth was seen with prolonged treatment of colo-205 with extended dosing on either schedule (Fig. 4d), indicating acquired resistance did not develop over the period of treatment.

**Fig. 5:**
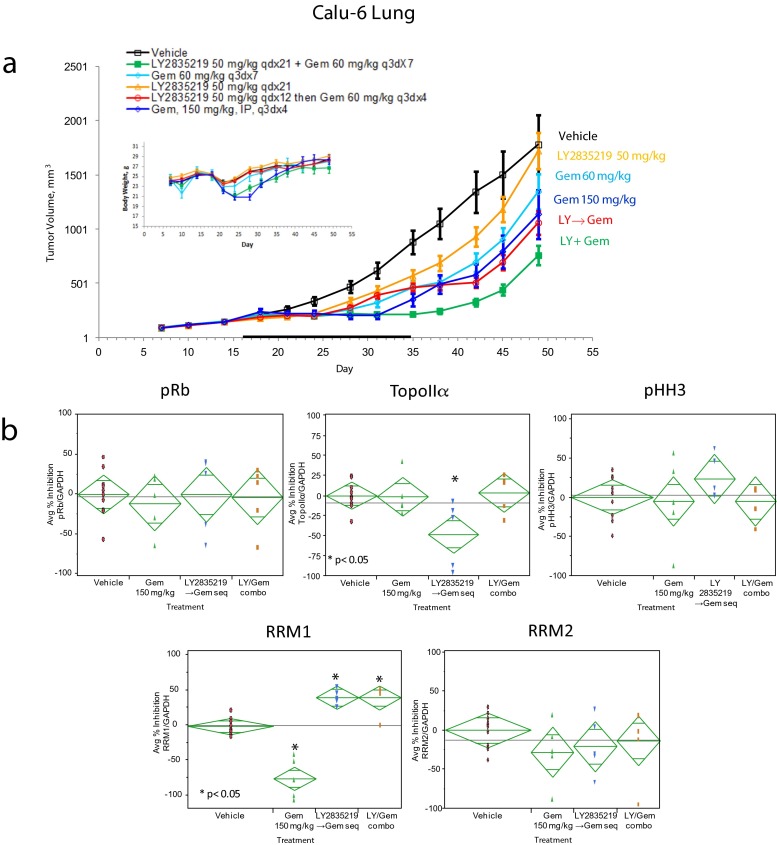
In vivo antitumor activity of LY2835219 and gemcitabine. **a** Calu-6 lung xenografts were treated with LY2835219 orally, with gemcitabine by intraperitoneal injection, LY2835219 followed by gemcitabine, or both compounds together. treatment period is indicated by horizontal black bar along the X-axis and body weight is shown in the upper left corner. **b** results of target inhibition studies

Cytotoxic drugs are commonly used to treat cancer; combining CDK4/6 inhibitors with cytotoxic drugs may increase their clinical effectiveness. Previous studies suggested some CDK inhibitors may antagonize cytotoxic efficacy when combined with cytotoxic drugs; therefore, require sequential administration [30]. We evaluated sequential and combination dosing of LY2835219 and the cytotoxic drug gemcitabine, an inhibitor of DNA synthesis approved for the treatment of several cancers, including pancreatic, metastatic breast, ovarian and non-small cell lung cancer. Calu-6 lung tumor xenografts were used as their response to gemcitabine has been previously characterized [31]. Individual doses of 50 mg/kg LY2835219, 60 or 150 mg/kg gemcitabine, a sequence of LY2835219 followed by gemcitabine or the 2 compounds together were given. Both LY2835219 and gemcitabine alone were active in inhibiting tumor growth; the combination gave a greater inhibition of tumor growth than either treatment alone (Fig. 5a). The sequenced group was also active, although not significantly different from the individual compounds, or the 150 mg/kg gemcitabine group. Together, the sequence and combination treatments indicate there was no antagonism. Comparison of changes in body weight for all groups (including vehicle) showed a transient weight loss upon initiation of dosing, followed by recovery to baseline, indicating no significant toxicity when LY2835219 and gemcitabine were administered together. Based on the available exposure data, there is no evidence of drug-drug interaction for any of the two agents used in this combination study. The mechanism of action for the antitumor activity of LY2835219 and gemcitabine was further analyzed by western blot analysis in tumors collected 24 h after the last dose. No significant changes were found for p-Rb, TopoIIα, or pHH3 except for an increase in TopoIIα in the sequence group (Fig. 5b). These results indicate a cell cycle arrest did not result from the treatments; the increase in TopoIIα seen for the sequenced treatment may indicate a partial cell cycle synchronization by LY2835219, resulting in an increase of cells in S phase. Expression of RRM1 and RRM2 was also analyzed. A significant increase of RRM1 was seen with the 150 mg/kg gemcitabine dose (75 % compared to vehicle), possibly due to cellular compensation resulting by its inhibition by gemcitabine. A significant inhibition (approximately 40 %) was seen for both the sequence and combination treatments, suggesting that inhibition of RRM1 expression by LY2835219 may be sensitizing tumor cells to the activity of gemcitabine.

## Discussion

The Rb pathway is dysregulated in more than 80 % of human cancers, highlighting its critical role in controlling cellular proliferation [4]. Pathway alterations found in human tumors include overexpression of D-type cyclins, activating mutations and amplification of CDK4, and Rb and p16/ink4a inactivation by deletion, mutation, or epigenetic silencing. Data for the role of the Rb pathway in primary human tumors are supported by extensive functional analysis in genetically engineered mouse models [32]. The importance of the pathway in tumorigenesis and recent success developing small molecule kinase inhibitors, make CDK4/6 a highly validated cancer drug target [33, 34]. Until recently, CDK inhibitors have shown limited activity in clinical trials because of issues with potency, selectivity and poor pharmacological/physiochemical properties. CDK4/6 inhibitors with improved pharmacokinetic and pharmacodynamic properties have recently been described and are now entering advance stages of clinical development. These recent results support the clinical potential for selective targeting of CDK4/6 in the treatment of cancer [35].

We describe the identification and preclinical characterization of LY2835219, a potent inhibitor of CDK4/6 with antiproliferative activity in a variety of tumor models in vitro and in vivo. In addition to monitoring p-Rb, we phenotypically assessed the cell cycle status in cellular and in vivo assays; as we previously showed p-Rb alone was not considered sufficient to determine cell cycle activity [36]. In vitro LY2835219 inhibits Rb phosphorylation by CDK4/6, resulting in a sustained G1 arrest at concentrations 50-fold greater than the cellular IC_50_ for p-Rb inhibition, indicating no off-target activity. Consistent with the cell cycle mechanism of action of LY2835219, cell cycle arrest occurred only in Rb-proficient cells. Cell cycle activity was further tested in vivo by monitoring markers for specific cell cycle compartments. LY2835219 effectively inhibited phosphorylation of Rb at serine 780, and significant inhibition of all markers was achieved in colo-205 xenografts at doses of 12.5 mg/kg and higher, indicating a block at the G1 restriction point preventing cells from cycling into the S and M compartments (monitored by TopoIIα and pHH3, respectively). Maximum inhibition of p-Rb and the cell cycle occurred 24 h after LY2835219 administration and was sustained after maximal plasma exposure (Fig. 3). Release from the G1 block was seen through the temporal repopulation of S and M phases, indicated by the sequential loss of p-Rb inhibition (indicating active CDK4/6, and cells entering late G1 phase) followed by TopoIIα (S phase), and finally pHH3 (M phase). In colo-205 xenograft dose response target inhibition studies the TED_70_ for LY2835219 is 14 mg/kg for in vivo inhibition of both pRb and TopoII (Fig. 3a). This value is in agreement with the observed minimally efficacious dose of 12.5 mg/kg for tumor growth inhibition. Due to the cell cycle-dependent nature of the PD effect and the observed indirect PK/PD relationship in mouse models, plasma concentrations do not directly correlate with in vivo target inhibition in xenografts, as maximal target inhibition is observed at 24 h post-acute oral dose (Fig. 3b). An integrated and semi-mechanistic pharmacokinetics/pharmacodynamics (PK/PD) model was developed to describe the indirect PK/PD relationship between LY2835219 plasma concentration, inhibition of pRb and subsequent cell cycle arrest, and in vivo efficacy in colo-205 bearing mice [37]. This PK/PD analysis concludes that sustained, continuous in vivo pRb inhibition and cell cycle arrest are required for robust efficacy in colo-205, and supports a continuous dosing strategy achieving minimum steady state trough plasma concentrations of approximately 200 ng/mL (approximately 400 nM). In this context, it is important to note that the desired cell cycle phenotype in preclinical models in vitro (Fig. 2) and in vivo (Fig. 3) is retained when LY2835219 is dosed up to single digit micromolar concentrations (Fig. 3b). In a phase 1 study in advanced cancer patients, pharmacodynamic activity as well as clinical efficacy were observed. Plasma concentrations in this study reached a mean maximum steady state value of 562 nM [38, 39], consistent with the target plasma concentrations from our PK/PD model. In total, these data suggest that, in vivo, LY2835219 inhibits CDK4/6 at plasma concentrations clinically achievable, resulting in a G1 arrest that can be sustained with continuous oral dosing.

CDK4 and CDK6 are functionally redundant and ubiquitously expressed, requiring inhibition of both targets to inhibit proliferation of tumors arising from different cellular origins. LY2835219 is a potent inhibitor of both CDK4 and CDK6 kinases with potent cellular inhibition of pRb (Fig. 2a) of approximately 100 nM and has good selectivity over other closely related kinases (approximately 1000-fold selective for CDK1 and ≥ 100-fold selective for CDK2 and CDK5). Biochemical kinase profiling is best interpreted in the context of the functional cellular potency and selectivity for a given drug candidate (see Table 1 for cellular profiling). Factors to consider are the expected potency shift due to the higher intracellular ATP concentration and Km, conformational differences in the native state of protein kinases with respect to conditions and constructs used for biochemical assays, and compensation by other kinases not seen in biochemical assays. For LY2835219 among other kinases in the CMGC kinome family, some activity below 100 nM was observed against CDK9 and PIM1 kinases in biochemical assays. However, the inhibition of CDK9 seen in biochemical assays did not translate into cellular CDK9 activity (inhibition of pCTD and a G2/M arrest in U2OS cells, Figure S1). Similarly, while activity against PIM1 was seen in biochemical assays, monitoring of the cell cycle in vitro and in vivo shows LY2835219 induces a G1 cell-cycle arrest, even in MV4-11 and Jeko-1 cells and xenografts, which express high levels of PIM1 [40–43]. In MV4-11 cells and unlike PD0332991, LY2835219 showed significant inhibition of pBAD (Ser112), and p4E-BP1 (Thr37 and 46), known substrates of PIM kinases, at concentrations equal or higher than 625 nM after 24 h of incubation (Figure S3). Both compounds showed similar potent inhibition of pRb (Ser780) in MV4-11. Although the presence of functional cellular PIM1 activity for LY2835219 in these hematological tumor types that are presumably sensitive to PIM inhibition seems possible, the very similar MV4-11 and Jeko-1 xenograft efficacy observed for this molecule with respect to another CDK4/6 inhibitor without PIM1 activity (Fig. 4c, Table 2) suggests a modest effect of this activity compared to CDK4/6 for this particular tumor type, possibly due to compensation by PIM2 or other kinases. More work is be needed to further elucidate the potential role of this PIM1 activity in other tumor types. Overall, these results demonstrate that, in MV4-11, inhibition of PIM1 does not abrogate G1 cell-cycle arrest that arises from the inhibition of CDK4/6.

In vivo target inhibition studies showed LY2835219 produced a cell cycle arrest 24 h after dosing, and was reversible (Fig. 3). The arrest was maintained even after peak plasma concentrations were achieved and indicates target inhibition and antitumor activity can be maintained with daily dosing schedules. This was confirmed in efficacy studies. Significant antitumor activity was seen when LY2835219 was administered orally in several different human tumor xenografts. In colo-205, doses of 12.5 mg/kg and higher inhibited tumor growth and were associated with inhibition of p-Rb, TopoIIα, and pHH3. This shows in vivo antitumor activity results from inhibition of CDK4/6 resulting in a G1 arrest. The antitumor activity of LY2835219 was confirmed in several additional human xenografts representing different human histologies, and was dose-dependent. LY2835219 antitumor activity was further confirmed by comparison of in vivo efficacy to another CDK4/6 inhibitor, PD0332991 (which has similar activity against CDK4/6). In all models tested, both compounds gave similar antitumor activity at similar doses (Fig. 4, Table 2). Finally, antitumor activity of LY2835219 was sustained for up to 56 days with continuous or intermittent dosing schedules in mice bearing colo-205 xenografts without significant adverse events or evidence of acquired resistance (Fig. 4d).

LY2835219 can be combined with the cytotoxic drug gemcitabine, approved to treat several types of tumors, including lung cancer [44]. In calu 6 lung xenografts both LY2835219 and gemcitabine alone were active in inhibiting tumor growth. Administering the compounds together or in sequence had greater antitumor activity than the individual treatments and was equivalent to the maximum tolerated dose of gemcitabine (150 mg/kg) [31]. Contrary to other xenograft models, neither inhibition of p-Rb nor a cell cycle arrest was associated with the antitumor activity of LY2835219 in calu-6. However, a significant inhibition of RRM1 was seen when LY2835219 was combined with gemcitabine. In vitro and in vivo chemosensitivity to gemcitabine strongly correlates to RRM1 expression [45, 46], consistent with the modulation of RRM1 seen for LY2835219 and gemcitabine in our experiments. CDK4/6 can phosphorylate substrates other than Rb, such as FOXM1 and E2F1 [10, 11], which may explain the inhibition of RRM1 and the absence of inhibition of p-Rb and a cell cycle arrest. Alternatively, LY2835219 in calu-6 cells may result in a low level of p-Rb inhibition not detected by western blot or leading to a cell cycle arrest, but is sufficient to inhibit the expression of RRM1. Consistent with the inhibition of RRM1 in our experiments, expression of RRM1 was previously reported to be highly sensitive to regulation by p-Rb [28]. Previously reported data that PD0332991 has efficacy in vivo without inhibition of p-Rb [47] also suggest low levels of inhibition of p-Rb may lead to antitumor activity in vivo. Significantly, H460 contains activating KRAS mutation in codon 61, like calu-6, and shows an attenuated response to LY2835219 (25 mg/kg dose Table 2), consistent with a role for KRAS modifying response to CDK4/6 inhibitors [48, 49].

In conclusion, we describe the identification and characterization of LY2835219, a potent inhibitor of CDK4/6. Broad antitumor activity was observed in human xenograft tumors of diverse histologic origin that represent human cancers with alterations in the Rb pathway, including MCL, colorectal, lung, glioblastoma and AML. The antitumor activity results predominantly from a G1 cell cycle arrest that correlates with inhibition of p-Rb. In calu-6 lung xenograft tumors, LY2835219 can be combined with gemcitabine resulting in additive antitumor activity and inhibition of RRM1 in the absence of a cell cycle arrest. These results indicate that the antitumor activity of CDK4/6 inhibitors can be context-sensitive, as previously reported in genetically engineered mouse models for glioma and lung cancer [47, 48]. In addition to its broad antitumor activity, LY2835219 has several additional desirable properties. Efficacy is correlated with tumor Rb status and in vivo target inhibition, allowing for the development of biomarkers for patient stratification and to monitor drug response. A unique feature of LY2835219 is its ability to effectively cross the blood–brain barrier; it has been shown to be active in orthotopic brain tumor xenografts alone or in combination with temozolomide (manuscript in preparation). The Rb pathway is dysregulated in approximately 78 % of glioblastoma and in tumors that commonly metastasize to the brain such as breast, lung, and melanoma [49, 50]. These properties support further clinical study of LY2835219 in the treatment of both brain metastases and tumors arising in the central nervous system [51–53]. LY2835219, is currently in clinical trials (ClinicalTrials.gov identifiers: NCT01394016, NCT01739309, NCT02079636, NCT02057133, NCT02117648, NCT02014129).

## Electronic supplementary material

Below is the link to the electronic supplementary material.

ESM1 (DOC 271 kb).
