# Supplementary material for: Preclinical characterization of the CDK4/6 inhibitor LY2835219: in-vivo cell cycle-dependent/independent anti-tumor activities alone/in combination with gemcitabine
Source: Invest New Drugs. 2014 Jun 13;32(5):825–37. doi: 10.1007/s10637-014-0120-7 (PMC4169866; doi:10.1007/s10637-014-0120-7)
Supplement: Supplementary file 1 — (DOC 271 kb). [file 10637_2014_120_MOESM1_ESM.doc]

**SUPPLEMENTAL METHODS**

**Title: *Preclinical characterization of the CDK4/6 inhibitor LY2835219: in-vivo cell cycle-dependent/independent anti-tumor activities alone/in combination with gemcitabine***

**Journal Name:** Investigational New Drugs

**Authors:** ^1*^Lawrence M. Gelbert, ^1^Shufen Cai, ^1^Xi Lin, ^2^Concepcion Sanchez-Martinez, ^2^Miriam del Prado, ^2^Maria Jose Lallena, ^2^Raquel Torres, ^1^Rose T. Ajamie, ^3^Graham N. Wishart, ^4^Robert Steven Flack, ^4^Blake Lee Neubauer, ^4^Jamie Young, ^1^Edward M Chan, ^1^Philip Iversen, ^3^Damien Cronier, ^1^Emiko Kreklau, ^1^Alfonso de Dios

**Authors’ Affiliation:** ^1^Eli Lilly and Company, Lilly Corporate Center, Indianapolis, IN 46285, ^2^Eli Lilly and Company, Alcobendas (Madrid), Spain, ^3^Eli Lilly and Company, Windlesham, United Kingdom. ^4^Covance Laboratories, Greenfield, IN 46140.

**Corresponding Author:** Alfonso de Dios. Email: de_dios_alfonso@lilly.com

**CDK4 Inhibition Assay**

Ten μL of test compound in 20% DMSO, 20 μL of adenosine 5'-triphosphate (ATP) and C-Terminal Retinoblastoma Fragment (CTRF) (Upstate cat# 12-439) solution, and 10 μL of enzyme solution are mixed in a 96-well plate. The ATP and CRTF solution is prepared from a mixture of 40 μM ATP, 0.16 μCi [^33^P] –ATP, and 1μM CTRF diluted in kinase buffer of 68 mM 4-(2-hydroxyethyl)-1-piperazineethanesulfonic acid (HEPES) pH 7.4, 6.72 mM MgCl_2_, 6.72 mM dithiothreitol (DTT), and 0.013% TRITON^TM^ X-100. The enzyme solution is prepared from 8 ng CDK4 enzyme (Proqinase) diluted in the kinase buffer described above. Test compounds are serially diluted 1:3 in 20% DMSO to create a 10-point curve at a starting concentration of 20 μM. Twenty percent DMSO buffer alone without added test compound is employed as a control, 500 mM ethylene diamine tetraacetic acid (EDTA) is used to determine the level of background ^33^P in the absence of enzyme activity. Reagents are mixed and incubated for 90 min at 20 ºC. The reaction is terminated by the addition of 80 μL 10% (v/v) H_3_PO_4_ and precipitation of material onto glass fibre filter plates (Millipore, MAFC N0B 50). The wells are washed 4 times with 0.5% H_3_PO_4_ and the radioactivity incorporated is determined with a microplate scintillation counter (Microbeta Trilux, Wallac).

The difference between the median value of high and low control is taken as 100% activity. A 4-parameter logistic curve fit is used to generate the IC_50_ values using ActivityBase™ software (IDBS, Alameda CA).

**CDK6 Inhibition Assay**

Ten μL of test compound in 20% DMSO, 20 μL of ATP and CTRF (Upstate cat# 12-439) solution, and 10 μL of enzyme solution are mixed in a 96-well plate. The ATP and CRTF solution is prepared to give a final concentration of 100 μM ATP, 0.5 μCi [^33^P] –ATP, and 0.8 μM CTRF diluted in kinase buffer of 68 mM HEPES pH 7.4, 6.72 mM MgCl_2_, 2.64 mM DTT, and 0.004% TRITON^TM^ X-100. The enzyme solution is prepared for a final concentration of 1.7 ng/μL CDK6 enzyme (Proqinase) diluted in the kinase buffer described above in the CDK4 inhibition assay. Test compounds are serially diluted 1:3 in 20% DMSO to create a 10-point curve at a starting concentration of 20 μM. Twenty percent DMSO buffer alone without added test compound is employed as a control, 500 mM EDTA is used to determine the level of background ^33^P in the absence of enzyme activity. Reagents are mixed and incubated for 90 min at 20 ºC. The reaction is terminated by the addition of 80 μL 10% (v/v) H_3_PO_4_ and precipitation of material onto glass fiber filter plates (Millipore, MAFC N0B 50). The wells are washed 4 times with 0.5 % H_3_PO_4_ and the radioactivity incorporated is determined with a microplate scintillation counter (Microbeta Trilux, Wallac). The data is analyzed in the same manner as for CDK4.

**Assay for inhibition of PIM1 Kinase**

Pim-1 (human, 0.46 nM final concentration) is incubated with 8 mM MOPS pH 7.0, 0.2 mM EDTA, 100 µM KKRNRTLTV, 10 mM MgAcetate and [γ-^33^P-ATP] (specific activity approximately 500 cpm/pmol, concentration as required). The reaction is initiated by the addition of the MgATP mix and then incubated for 40 minutes at room temperature. The reaction is stopped by the addition of 3% phosphoric acid solution. Ten µL of the reaction is then spotted onto a P30 filtermat and washed 3 times for 5 minutes in 75 mM phosphoric acid, and once in methanol prior to drying and scintillation counting. For compound inhibition testing, compounds provided as 10 mM stocks in 100% DMSO are diluted 1:10 in 100% DMSO to give a 50x stock of the top concentration of the curve. The 50x stock is then serially diluted 1:3 in 100% DMSO to create a 10-point concentration-response curve and diluted 1:50 (20 µM to 0.001 µM final in 2% final DMSO concentration) in the reaction mixture to determine compound activity. Control wells contain DMSO only while the baseline is established in control wells acid-stopped immediately. The percent inhibition determined from the controls on each plate and 10-point compound concentration data were then fit to a 4-parameter logistic equation using ACTIVITYBASE 4.0.

**Enzyme kinetic studies.**

**CDK4/cyclinD1 Enzyme Kinetic Assays**. CDK4 activity was measured by the phosphorylation of CTRF using [γ-33P] ATP at 20 °C in a 96 well plate filtration assay format. The enzyme reaction was conducted in final a volume of 25 μL in a kinase buffer containing 68 mM Hepes at pH 7.5, 6.72 mM MgCl2, 6.72 mM dithiothreitol (DTT), and 0.013% TRITONTM X-100. 10uL of CDK1/cyclinD1 enzyme (3.5 nM final concentration in the assay) was added onto a mix of substrate and test compound. Concentration of compounds will be ranged from 5 μM to 0.3 nM and ATP will vary from 1mM to 7.8 μM (1.12 mCi [^33^P] –ATP per μmol ATP) and a constant concentration of 0.8 μM C-Terminal Retinoblastoma Fragment (CTRF). Reaction will be carried out in a 96-well plate. 4% (v/v) DMSO buffer without test compound is used as a control to obtain ATP saturation curve and KMATP. 125 mM ethylene diamine tetraacetic acid (EDTA) is used to determine the level of background 33P in the absence of enzyme activity. Reagents are mixed and incubated for 50 min at 20 ºC. The reaction is terminated by the addition of 80 μL 10% (v/v) H3PO4 and precipitation of material onto glass fibre filter plates (Millipore, MAFC N0B 50). The wells are washed 4 times with 0.5% H3PO4 and the radioactivity incorporated is determined with a microplate scintillation counter (Microbeta Trilux, Wallac).

**CDK6/cyclinD1 Enzyme Kinetic Assays**. CDK6 activity was measured by the phosphorylation of CTRF using [γ-33P] ATP at 20 °C in a 96 well plate filtration assay format as is also described for CDK4/cyclinD1. The enzyme reaction was conducted in final a volume of 25 μL in a kinase buffer containing 68 mM Hepes at pH 7.5, 6.72 mM MgCl2, 2.7 mM dithiothreitol (DTT), and 0.004% TRITONTM X-100. 10uL of CDK6/cyclinD1 enzyme (7 nM final concentration in the assay) was added onto a mix of substrate and test compound. Concentration of compounds will be ranged from 5 μM to 0.3 nM and ATP will vary from 1mM to 7.8 μM (1.12 mCi [33P] –ATP per μmol ATP) and a constant concentration of 0.8 μM C-Terminal Retinoblastoma Fragment (CTRF). Reaction will be carried out in a 96-well plate. 4% (v/v) DMSO buffer without test compound is used as a control to obtain ATP saturation curve and KMATP. 125 mM ethylene diamine tetraacetic acid (EDTA) is used to determine the level of background 33P in the absence of enzyme activity. Reagents are mixed and incubated for 50 min at 20 ºC. The reaction is terminated by the addition of 80 μL 10% (v/v) H3PO4 and precipitation of material onto glass fibre filter plates (Millipore, MAFC N0B 50). The wells are washed 4 times with 0.5% H3PO4 and the radioactivity incorporated is determined with a microplate scintillation counter (Microbeta Trilux, Wallac).

**Kinetic data analysis.** Data obtained were fitted to the Michaelis-Menten equation for a competitive inhibitor [1] using GraphPad Prism. After representing double reciprocal plot, Ki values were calculated from the re-plots of the slopes versus the [I] where -Ki will correspond to the X-intercept of the regression line. Data reported are the resulting of averaged values obtained in independent duplicate experiments.

[1]
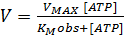
; where
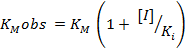


**Cellular assay for inhibition of Phosphorylation of Retinoblastoma Protein (p-Rb) and DNA Content Assay**

COLO 205 cells from the American Type Culture Collection (ATCC) are plated at 2000 cells/well in 96-well Beckman Dickinson BIOCOAT™ plates and incubated in RPMI 1640 medium (e.g., GIBCO, catalog # 52400-025) with 10% Fetal Bovine Serum (FBS, e.g., Gibco cat #11000-144 ) and 1 % sodium pyruvate (Gibco catalog #11360-070) in 37 °C, 5 % CO_2_ for 24 hours. Cells are treated by adding test compound to the medium, dosing at 10 points of 1:3 dilutions across the range of 20 µM to 0.001 µM, and with final DMSO concentration at 0.25 %. After 24-hour exposure to the compounds, cells are fixed with the PREFER™ fixative [Anatech LTD., Cat # 414] for 30 min at RT, then permeabilized with 0.1% TRITON® X100 in phosphate buffered saline (PBS) solution for 15 minutes at RT. Cells are washed twice with PBS, then digested with 50 µg/mL RNAse (Ribonuclease A, Sigma cat# R-6513) in a 37 °C incubator for 60 minutes. Fixed cells are blocked with 1% bovine serum albumin (BSA, Amersham cat # RPN412V) for 30 minutes. Primary antibody, anti-phosphoRB purified mouse monoclonal antibody (BD Pharmigen ca t# 558385), is added at 1:2000 in PBS with 1% BSA to the cells and incubated overnight at 4 °C. After 3 PBS washes, cells are incubated with Alexa488 labeled secondary antibody, goat anti mouse IgG Alexa 488 (Invitrogen cat # A11017) for 1 hour at RT. Again, they are washed 3 times with PBS, then 15 µM propidium iodide (1:100 dilution with PBS from the original solution, Invitrogen cat # P3566) is added to stain nuclei. Fluorescence plates are scanned with ACUMEN EXPLORER™ [Laser-scanning fluorescence microplate cytometer (comprising of 488 nm argon ion laser excitation and multiple photomultiplier tube detection), manufactured by TTP LABTECH LTD] to measure phosphorylation of Rb protein and DNA content. Image analysis is based on cellular fluorescent signals for identifying cells in different subpopulations. Assay outputs are a percentage of each identified subpopulations; percent phosphoRB positive, percent 2 N, and percent 4 N. The IC_50_ and EC_50_ values are determined by curve fitting to a 4-parameter logistic for each output using ACTIVITY BASE™.

**Cellular assay for inhibition of RNA polymerase CTD (Ser2) phosphorylation (pCTD)**.

U20S cells are cultured in McCoy’s 5A modified media (Invitrogen), supplemented with 10% FBS and plated in 96-well, flat-bottom plates at a density of 5,000 cells/well for incubation overnight. Compounds in DMSO stock solution are diluted serially 1:3 in McCoy’s 5A media and incubated with cells for 16 hours for multiplexing pCTD/DNA content readouts. Cells are fixed with Prefer (Anatech LTD, MI) for 30 minutes at room temperature and permeated with 0.1% Triton-X 100 in PBS or cold methanol (-20°C) for 15 minutes, respectively. After blocking with 1% BSA/PBS for 30 min, cells are incubated with primary antibody (CTD p-Ser2, Covance) at 1:1000 dilution in 1% BSA/PBS containing 50 ug/ml RNAse (R6513, Sigma) at 4°C overnight. Cells are then washed 3 times with PBS containing 0.1% tween-20 and incubated with the secondary antibody (Goat anti-mouse IgM Alexa 488, 1:2000 dilution) for 1 hour at room temperature, then stained with propidium iodide (Sigma) before being analysed on a Acumen Explorer™ Laser-scanning fluorescence microplate cytometer (TTP LabTech LTD, UK)) for pCTD and DNA content. The IC_50_ and EC_50_ values are determined by curve fitting to a 4-parameter logistic for each output using ACTIVITY BASE™.

Flow cytometry was used to determine cell cycle distribution in cell lines. Cells were cultured in growth media under subconfluent conditions and treated with indicated concentrations of compound solubilized in DMSO for 24 hours at 37°C/5% CO_2_. After treatment, cells were collected, washed once with 4° C PBS, and fixed with 70% ethanol/30% water at -20ºC for 30 minutes. Cells were then collected by centrifugation, washed once with PBS, and stained for 30 minutes at room temperature by the addition of 1 mL of propidium iodide/Triton X-100 staining solution (20µg/mL of propidium iodide, 200µg/mL DNase-free RNase A, 0.1%(v/v) Triton x-100). Cell cycle analysis was performed using a Cytomics™ FC500 (Coulter); data were analyzed using ModFit v3.1 software (Verity).

**Western blots**

The following primary antibodies were used: mouse anti-phospho retinoblastoma serine 780 (p-Rb, BD Pharmingen), rabbit anti-topoisomerase IIα (TopoIIα, Santa Cruz Biotechnology), rabbit anti-phospho histone H3 serine 10 (pHH3, Millipore Upstate Biotechnology), mouse anti- Glyceraldehyde-3-phosphate dehydrogenase (GAPDH, Meridian Life Science, Biodesign), mouse anti-dihydrofolate reductase (DHFR, Santa Cruz Biotechnology), mouse anti-ribonucleotide reductase M1 (RRM1, Chemicon), goat anti-ribonucleotide reductase M2 (RRM2, Santa Cruz Biotechnology), rabbit anti-phospho Bad serine 112 (pBAD, Cell Signalling), rabbit anti-phospho 4E-BP1 threonine 37/46 (p4E-BP1 Cell Signalling).

**Table S1 and Figure S1.** Compounds were counter-screened for cellular CDK9 activity using a high content assay in U2OS osteosarcoma cells. The assay detects CDK9 activity through both the inhibition of phosphorylation ser2 of the C-terminal repeat domain (pCTD) of RNA polymerase II, and a CDK9-modulated G2/M arrest [accumulation of cells with 4N DNA content] . The U2OS assay was validated with several CDK9 inhibitors, including flavopiridol and SNS-032 .

In our biochemical assays, flavopiridol inhibits CDK4 and CDK9 with IC_50_s of 40 nM and 11 nM in biochemical kinase assays, respectively and is consistent with previously published results . In our cellular assays, flavopiridol shows p-Rb inhibition in Colo205 with a relative EC_50_ of 55 nM. However, despite the potent p-Rb inhibition, a G1 arrest was not observed for flavopiridol in Colo205 (increase in cells with 2N DNA content, EC_50_ >20 μM, Table S1). In U2OS cells, flavopiridol inhibits pCTD (IC_50_=147 nM n=1, see Figure S1 for a representative run) along with an accumulation of cells in G2/M (increase in cells with 4N DNA content, Table S1), the cellular phenotype for inhibition of CDK9 .

Conversely, in colo-205 cells LY2835219 shows both inhibition of p-Rb and a corresponding G1 arrest, with relative EC_50_ of 72 nM for 2N cell population increase (Table S1 and manuscript Figure 2), indicating inhibition of CDK4/6. In the U2OS assay, LY2835219 shows a modest micromolar inhibition of pCTD (3.5 μM n=4 with SE of ± 1.6 μM, see Figure S1 for a representative run) which is within acceptable range for normal variability of a biochemical assay that typically will have a minimum significant ratio or MSR ≤3. This modest activity does not correspond to a significant CDK9 functional effect as indicated by no increase in the 4N cell population (Table S1, below). Additionally, in U2OS an increase in the 2N population was seen with LY2835219, indicating inhibition of CDK4/6. Based on these data, and comparison of LY2835219 to another CDK4/6 inhibitor showing similar antitumor activity (PD0332991, see manuscript Figure 4 and Table 2), we conclude the CDK9 activity observed in biochemical assays does not translate to either cellular or in vivo activity.

**Table S1** Cell cycle effect of LY2835219 and flavopiridol as determined in colo-205 and U2OS phenotypic assays.

|  |  |  | **CDK4/6 inhibitor** | | | | **Flavopiridol** | | | |
| --- | --- | --- | --- | --- | --- | --- | --- | --- | --- | --- |
| **Colo-205 cells** |  | **Untreated** | **0.74 μM** | **2.2 μM** | **6.6 μM** | **Untreated** | | **0.74 μM** | **2.2 μM** | **6.6 μM** |
|  | 2N | 50% | 83% | 84% | 75% | 55% | | 55% | 54% | 50% |
|  | S | 2% | 0% | 0% | 1% | 6% | | 3% | 2% | 1% |
|  | 4N | 48% | 1% | 16% | 24% | 38% | | 42% | 44% | 49% |
| **U2OS cells** |  | **Untreated** | **0.74 μM** | **2.2 μM** | **6.6 μM** | **Untreated** | | **0.74 μM** | **2.2 μM** | **6.6 μM** |
|  | 2N | 47% | 67% | 56% | 44% | 48% | | 23% | 22% | 23% |
|  | S | 11% | 8% | 6% | 7% | 8% | | 4% | 4% | 5% |
|  | 4N | 40% | 26% | 37% | 49% | 43% | | 73% | 74% | 72% |

**Figure S1** Inhibition of pCTD in U2OS cells for LY2835219 (A) and flavopiridol (B). Results shown from one representative experiment.

**A**

**B**


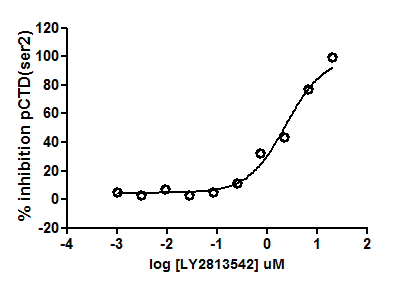

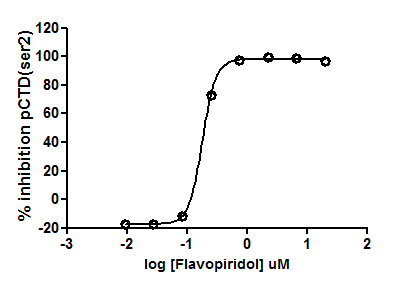


IC_50_= 3540 nmol/L

IC_50_= 177 nmol/L

**Figure S2.** Western blot analysis of MV4-11 cells treated with LY2835219. A dose-dependent decrease in the cell cycle markers p-Rb, topoisomerase IIα (TopoIIα) and pHH3, indicating a G1 cell cycle arrest corresponding to the DNA content analysis. Compound concentrations are μM.


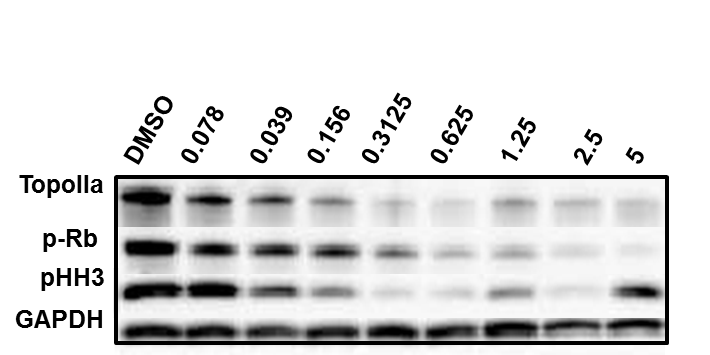


**Table S2.** Cell type, and relevant oncogenic mutations for cell lines described in the manuscript.

All cell types are Rb proficient except MDA-MB-468. Source: COSMIC database.

| **Cell line** | **Cancer** | **Mutation** |
| --- | --- | --- |
| **MCF10A** | Breast (Epithelial) | No major oncogenic mutations |
| **MDA-MB-361** | Breast (Luminal, ER+) | TP53, PI3KCA, ARID1 |
| **MDA-MB-231** | Breast (TNBC) | TP53, BRAF(G464V), KRAS, CDKN2A loss |
| **MDA-MB-468** | Breast (TNBC) | TP53, PTEN |
| **Colo-205** | Colorectal | TP53, BRAF, APC |
| **MV-4-11** | AML | FLT3-ITD |
| **U87-MG** | Glioblastoma | CDKN2A loss, PTEN |
| **NCI-H460** | Lung | KRAS, PIK3CA, CDKN2A loss |
| **Jeko1** | Mantle Cell Lymphoma | TP53, BRCA1, RB1 |
| **Calu-6** | Lung | TP53, KRAS |
| **U2OS** | Osteosarcoma | CCND2, CTNNB1 |
| **HCT116** | Colorectal | CTNNB1, KRAS, MET, NF1, PIK3CA |
| **A375** | Melanoma | BRAF |

**Figure S3.** Western blot analysis of MV4-11 cells treated for 24 hrs with LY2835219 and PD0332991. A significant inhibition of pBAD Ser(112) and p4E-BP1 Thr(37/46), markers related to inhibition of PIM kinases [5,6] was observed for LY2835219. A dose-dependent decrease in p-Rb was also observed. Compound concentrations are μM.


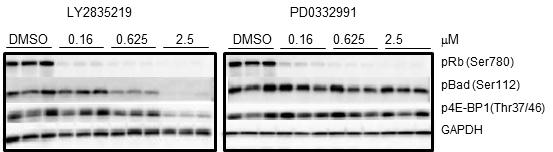


**References**

1. Torres-Guzman R, Chu S, Velasco JA, Lallena MJ. Multiparametric cell-based assay for the evaluation of transcription inhibition by high-content imaging. Journal of biomolecular screening. 2013;18:556-66.

2. Kaur G, Stetler-Stevenson M, Sebers S, Worland P, Sedlacek H, Myers C, et al. Growth Inhibition With Reversible Cell Cycle Arrest of Carcinoma Cells by Flavone L86-8275. Journal of the National Cancer Institute. 1992;84:1736-40.

3. Carlson BA, Dubay MM, Sausville EA, Brizuela L, Worland PJ. Flavopiridol Induces G1 Arrest with Inhibition of Cyclin-dependent Kinase (CDK) 2 and CDK4 in Human Breast Carcinoma Cells. Cancer Research. 1996;56:2973-8.

4. Chao SH, Fujinaga K, Marion JE, Taube R, Sausville EA, Senderowicz AM, et al. Flavopiridol inhibits P-TEFb and blocks HIV-1 replication. J Biol Chem. 2000;275:28345-8.

5. Keeton EK, McEachern K, Dillman KS, Palakurthi S, Cao Y, Grondine MS, et al. AZD1208, a potent and selective pan-Pim kinase inhibitor, demonstrates efficacy in preclinical models of acute myeloid leukemia. Blood 2014;123:905-13.

6. Yang Q, Chen LS, Neelapu SS, Miranda RN, Medeiros LJ, Ghandi V. Transcription and translation are primary targets of Pim kinase inhibitor SGI-1776 in Mantle Cell Lymphoma. Blood 2012;120:3491-00.
